# Cross-resistance of the pathogenic fungus *Alternaria alternata* to fungicides with different modes of action

**DOI:** 10.1186/s12866-019-1574-8

**Published:** 2019-09-02

**Authors:** Li-Na Yang, Meng-Han He, Hai-Bing Ouyang, Wen Zhu, Zhe-Chao Pan, Qi-Jun Sui, Li-Ping Shang, Jiasui Zhan

**Affiliations:** 1 Key Lab for Biopesticide and Chemical Biology, Ministry of Education, Fujian Agriculture and Forestry University, Fuzhou, China; 2 Fujian Key Laboratory of Plant Virology, Institute of Plant Virology, Fujian Agricultural and Forestry University, Fuzhou, 350002 Fujian China; 3 Yunnan Academy of Agricultural Sciences, Industrial Crops Research Institute, Kunming, Yunnan China; 4 State Key Laboratory of Ecological Pest Control for Fujian and Taiwan Crops, Fujian Agriculture and Forestry University, Fuzhou, China; 5 Department of Forest Mycology and Plant Pathology, Swedish University of Agricultural Sciences, Uppsala, Sweden

**Keywords:** *Alternaria alternata*, Mode of action, Cross resistance, Fitness penalty, Detached leaf assay, Resistance mechanism

## Abstract

**Background:**

Cross-resistance, a phenomenon that a pathogen resists to one antimicrobial compound also resists to one or several other compounds, is one of major threats to human health and sustainable food production. It usually occurs among antimicrobial compounds sharing the mode of action. In this study, we determined the sensitivity profiles of *Alternaria alternata*, a fungal pathogen which can cause diseases in many crops to two fungicides (mancozeb and difenoconazole) with different mode of action using a large number of isolates (234) collected from seven potato fields across China.

**Results:**

We found that pathogens could also develop cross resistance to fungicides with different modes of action as indicated by a strong positive correlation between mancozeb and difenoconazole tolerances to *A. alternata*. We also found a positive association between mancozeb tolerance and aggressiveness of *A. alternata*, suggesting no fitness penalty of developing mancozeb resistance in the pathogen and hypothesize that mechanisms such as antimicrobial compound efflux and detoxification that limit intercellular accumulation of natural/synthetic chemicals in pathogens might account for the cross-resistance and the positive association between pathogen aggressiveness and mancozeb tolerance.

**Conclusions:**

The detection of cross-resistance among different classes of fungicides suggests that the mode of action alone may not be an adequate sole criterion to determine what components to use in the mixture and/or rotation of fungicides in agricultural and medical sects. Similarly, the observation of a positive association between the pathogen’s aggressiveness and tolerance to mancozeb suggests that intensive application of site non-specific fungicides might simultaneously lead to reduced fungicide resistance and enhanced ability to cause diseases in pathogen populations, thereby posing a greater threat to agricultural production and human health. In this case, the use of evolutionary principles in closely monitoring populations and the use of appropriate fungicide applications are important for effective use of the fungicides and durable infectious disease management.

**Electronic supplementary material:**

The online version of this article (10.1186/s12866-019-1574-8) contains supplementary material, which is available to authorized users.

## Background

Plant pathogens can have devastating effects on a wide range of food crops and are responsible for a number of pandemics causing catastrophic effects on social stability such as the Irish and Bengal famines [1]. In addition, mycotoxins synthesized by some pathogens such as *Aspergillus flavus* and *Fusarium verticillioides* can directly harm human and animal health [2]. Therefore, the effective management of plant pathogens is a paramount task for ensuring human health and social stability. The introduction of synthetic fungicides revolutionizes agricultural production both in quantity and quality by providing highly effective management of plant diseases [3, 4]. However, repeated and excessive application of same active compounds over large spatial scales can lead to the development of fungicide resistance in pathogen populations, rapidly rendering efficacy to manage plant diseases [5, 6].

Synthetic fungicides can be classified to site-specific and site non-specific (multisite) according to modes of action. Site-specific fungicides are highly active and often systemic (taken up by and distributed throughout plants), resulting in good disease mitigation at very low dose to specific fungal groups. Site non-specific fungicides, usually having a broader breadth of metabolic activity, can be used to prevent or eradicate a wider range of plant pathogens. Resistance to site-specific fungicides can occur as a result of changes in a single amino acid of the target protein in the pathogen, whereas for site non-specific fungicides the development of resistance involves multiple nucleotide changes across several targeted genes in the pathogen genome. Therefore, it is commonly believed that the risk of developing resistance to site non-specific fungicides is lower than that to site-specific fungicides [3]. However, mutations to fungicide resistance may generate fitness penalties that reduce the competitiveness of pathogens in the absence of the corresponding fungicide(s) as a result of the changes in DNA sequences impeding their important cellular and biochemical functions [7, 8]. Thus, appropriate implementation of fungicides in agricultural practices that minimizes directional selection on pathogen populations by mixing or rotating fungicides with different modes of action is thought to an effective approach to slowdown the development of field resistance.

Pathogen populations that develop resistance to one fungicide sometimes can simultaneously resist one or several other fungicides-a phenomenon known as cross-resistance. Usually, cross-resistance appears among fungicides with the same mode of action [9, 10]. For example, *Alternaria alternata* the causal agent of leaf blight of pistachio was reported to show cross-resistance to difenoconazole, propiconazole, and tebuconazole, all of these fungicides act through demethylation inhibition (DMI) [11]. However, cross-resistance may also occur between fungicides with distinct modes of action [12]. This is usually caused by limiting intercellular accumulation of active compounds through enhanced drug efflux, detoxification or reduced drug uptake. For example, the overexpression of efflux transporter genes made the grey mould fungus *Botrytis cinerea* simultaneously resistant to a broad-spectrum of fungicides [13]. In field isolates of *Zymoseptoria tritici*, enhanced efflux contributed to the pathogen’s cross-resistance to several fungicides with different modes of action [14]. In this study, we used efficacy profile of mancozeb and difenoconazole in *Alternaria alternata* from potato to test the development of cross-resistance to fungicides with different modes of action.

Mancozeb is a dithiocarbamate fungicide classified by the Fungicide Resistance Action Committee (FRAC) to mode-of-action group M (Multi-site Action). Mancozeb itself is not fungicidal, but the ethylene bisisothiocyanate sulfide (EBIS) and ethylene bisisothiocyanate (EBI) generated after its exposure to water and UV light are active toxicants which interfere with sulphydryl groups of enzymes involving at least six biochemical processes within cytoplasm and mitochondria of fungal cells [15]. On the other hand, difenoconazole, a 1, 2, 4-triazole, is a demethylation inhibitor (DMI) that targets sterol 14α-demethylase (CYP51), an important regulatory enzyme in the ergosterol biosynthetic pathway [16].

Mancozeb and difenoconazole are routinely used together to control plant diseases in many parts of the world. As a result, many studies have dedicated to understand their efficacy of mitigating disease epidemics and molecular mechanisms contributing to the development of mancozeb and difenoconazole resistance in pathogens [17, 18]. Although the risk of developing mancozeb resistance is low, substantial reduction in sensitivity to the fungicide has been documented in many pathogen species [15]. Genome-wide analysis reveals that this reduction in pathogen sensitivity is associated with the genes involving the formation of transcriptional machinery, cellular pH regulation, and multidrug transporters [17]. For difenoconazole, resistant phenotypes have been reported in many field populations of fungal pathogens [19, 20]. Up-regulation of ABC or MFS transporters to increase efflux, alterations of the *Cyp51* gene to decrease the affinity of DMIs for their target site and the raised levels of sterol 14α-demethylase caused by overexpression of the *Cyp51* gene are the three main mechanisms responsible for the final phenotype of difenoconazole resistance in plant pathogens [13]. However, information concerning the evolutionary interaction of resistance to the two fungicides in pathogens and how such interaction may impact the development of pathogen’s aggressiveness is limited but important to effectively administer fungicides for sustainable food production and social development.

Mancozeb and difenoconazole are also commonly used together to control potato early blight, a foliar disease forming dark-brown to black necrotic lesions with concentric rings [21]. The disease occurs worldwide but is most prevalent and severe in areas experiencing warm and alternating dry and high humidity periods [22]. Potato early blight can be caused by several species of *Alternaria* [23], but *A. alternata*, a smaller-spore species which produces conidia containing 8–12 spores with numerous secondary and occasionally tertiary chains branching from apical and median cells [24], is the main causal agent in China [25, 26]. No teleomorphs (sexual fruiting body) have yet been detected either in the field or under laboratory conditions, but population analyses of genetic variation, mating type distribution and phylogenetic trees all suggest that sexual reproduction or some other mechanism beyond mutation must occur to generate genetic variation of the pathogen for ecological adaptations [25]. High genetic variation in the pathogen also increases its potential to develop fungicide resistance, posing a great threat to the long-term management of the pathogen.

Thus, the specific objectives of this study were to: i) determine the spatial distribution of *A. alternata* sensitive to mancozeb and difenoconazole in China; ii) assess cross-resistance of mancozeb and difenoconazole sensitivity in *A. alternata*; and iii) evaluate whether there is a fitness cost associated with fungicide resistance by conducting correlation analyses of mancozeb and difenoconazole tolerances with pathogenicity in *A. alternata*.

## Results

### Frequency distribution of mancozeb and difenoconazole sensitivity in *A. alternata*

**Fig. 1:**
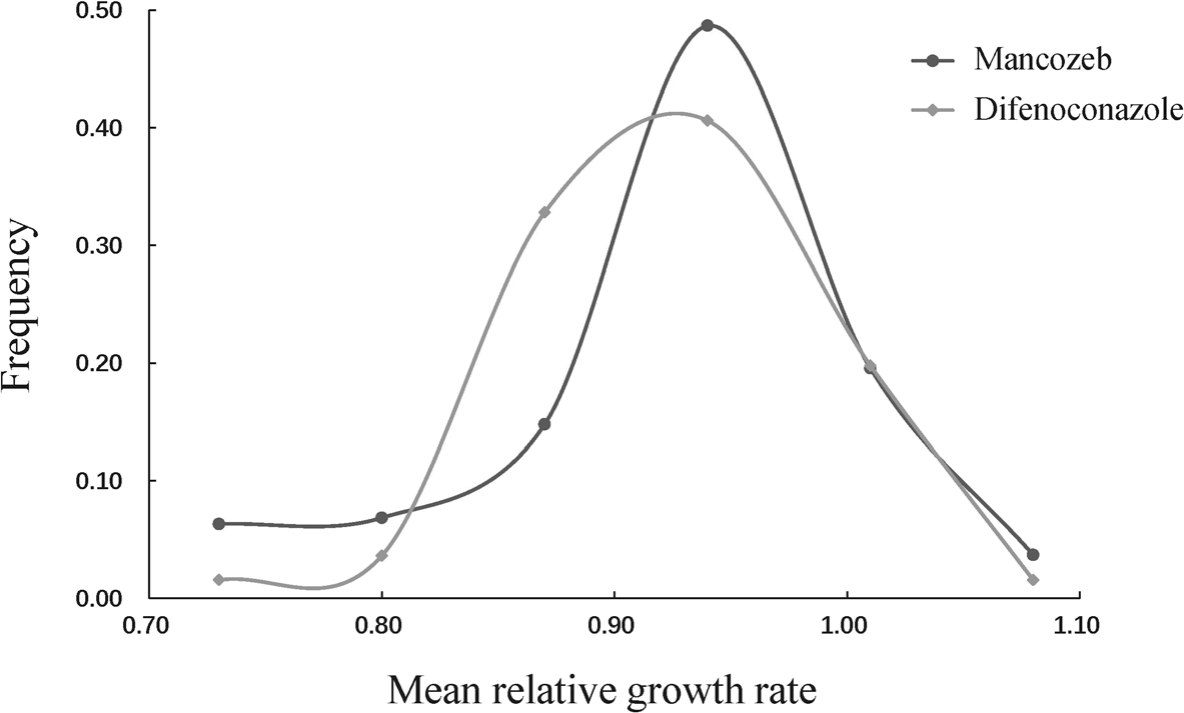
The frequency distribution of fungicide tolerance in the *Alternaria alternata* isolates collected from seven potato fields across China. Fungicides tolerance was measured with mean relative growth rate (MRGR) which calculated from the average of relative growth rates (RGRs) at three different fungicide concentrations

A total of 234 *A. alternata* isolates were used to measure mancozeb and difenoconazole tolerance. The mean relative growth rate (MRGR) calculated from the average of relative growth rate (RGR) at three fungicide concentrations was used to demonstrate the frequency distribution of mancozeb and difenoconazole tolerances. Frequency distributions of MRGRs in mancozeb and difenoconazole were visualized by grouping isolates into 6 bins differing by 0.07 units. Exploratory analyses revealed that these bin allocations yielded the best distribution with equal spacing between bin means. MRGR in both mancozeb and difenoconazole tolerances displayed a continuous and unimodal distribution with slight shifting to the right. The MRGR ranged from 0.63 to 1.07 with an average of 0.89 in mancozeb and 0.66 to 1.06 with an average of 0.89 in difenoconazole, respectively (Fig. 1). MRGR distributions of both mancozeb and difenoconazole shifted slightly to right (Additional file 1: Figure S1).

### Frequency distribution of aggressiveness of *A. alternata*

**Fig. 2:**
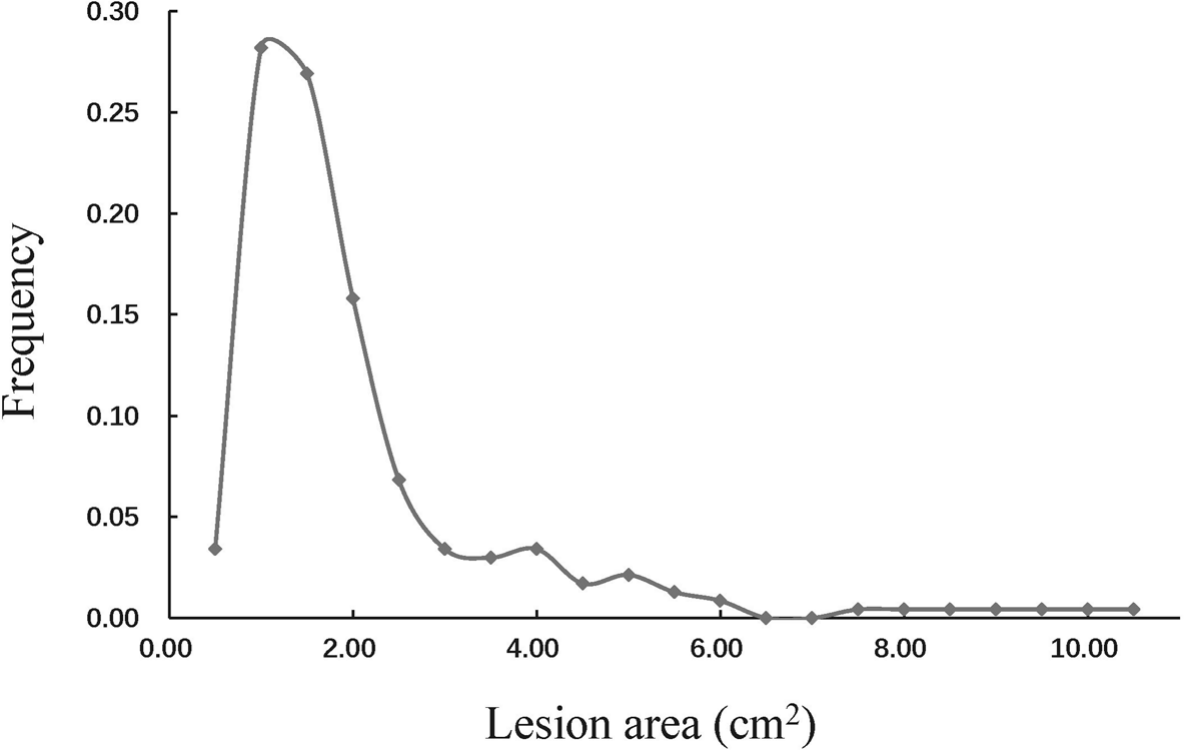
The frequency distribution of pathogenicity in the *Alternaria alternata* isolates. Pathogenicity was measured with lesion area at 5th days post inoculation on detached leaves of susceptible cultivar Favorita

Aggressiveness of *A. alternata* was measured as the lesion area at 5^th^ day after inoculation on detached leaves of the susceptible cultivar, Favorita. The frequency distribution of the lesion area was visualized by grouping isolates into 21 bins in 0.5-unit interval. This analysis revealed a wide variation in lesion area among the *A. alternata* isolates, ranging from 0.00 to 10.09 with an average of 1.84 cm^2^. The lesion area displayed a unimodal distribution peaking at 1.0 cm^2^, with a long tail stretching to high aggressiveness (Fig. 2). More than 70% isolates had a lesion area distributing between 0.5 and 2.0 cm^2^. Seven isolates had the lesion area > 7.5 cm^2^, suggesting their higher aggressiveness.

### Differences in fungicide tolerances and aggressiveness among *A. alternata* populations

Analysis of variance showed that “population” and “isolates” contributed significantly to the variation in aggressiveness, mancozeb tolerance and difenoconazole tolerance in *A. alternata* isolates sampled across China. “Concentration” also significantly contributed to the variation of mancozeb and difenoconazole tolerances (Table 1). “Mating type” contributed significantly to the variation of aggressiveness but not to fungicide resistance. Further LSD analysis showed that *A. alternata* populations exhibited similar trends of tolerance to mancozeb and difenoconazole. Populations HLJ and HNN were most tolerant to both mancozeb and difenoconazole while populations SDG, HBI and YNN were least tolerant to both fungicides. Populations FJN, HNN and YNN showed highest aggressiveness while populations SDG and HBI showed the lowest aggressiveness. Overall, population HNN exhibited both high aggressiveness and mancozeb and difenoconazole tolerances, while population SDG exhibited both low aggressiveness and fungicide tolerance (Table 2). On average, Mat-2 isolates showed significantly higher aggressiveness than Mat-1 isolates (Table 3), suggesting that mating type has an impact on the aggressiveness of the pathogen.

**Table 1:** Analysis of variance for the effect of mating type, isolate, population and fungicide concentrations on the pathogenicity and fungicide tolerance of *Alternaria alternata* as measured by lesion size at 5^th^ days post inoculation on detached potato leaves and the relative growth rate (RGR) of the isolates in the presence of difenoconazole or mancozeb at three concentrations to the absence of the fungicides

	DF	SS	MS	*F*	Pr > *F*
Lesion area					
Mating type	1	23.19	23.19	7.61	0.006
Population	6	124.54	20.76	6.81	< 0.0001
Isolate (population)	223	2333.46	10.46	3.43	< 0.0001
Error	606	1846.60	3.05		
Mancozeb tolerance					
Mating type	1	0.001	0.001	0.11	0.74
Population	6	0.64	0.11	9.13	< 0.001
Isolate (population)	194	12.52	0.07	5.54	< 0.001
Concentration	2	1.51	0.75	64.77	< 0.001
Error	1366	15.03	0.01		
Difenoconazole tolerance					
Mating type	1	2.52 × 10^−5^	2.52 × 10^−5^	0.00	0.96
Population	6	0.53	0.09	8.07	< 0.0001
Isolate (population)	187	5.97	0.03	2.94	< 0.0001
Concentration	2	1.27	0.64	58.64	< 0.0001
Error	1445	15.90	0.01

**Table 2:** Least significant difference test for lesion size, mean difenoconazole and mancozeb relative growth rates (RGRs) among the seven *A. alternata* populations collected from potato in China

Populations	Mancozeb RGR	Difenoconazole RGR	Lesion area
IMG	0.91^AB^	0.88^B^	1.92^BC^
FJN	0.91^BC^	0.91^A^	2.57^A^
HLJ	0.92^A^	0.91^A^	1.83^BC^
SDG	0.88^D^	0.86^B^	1.40^C^
HBI	0.88^CD^	0.87^B^	1.53^C^
HNN	0.92^A^	0.91^A^	2.18^AB^
YNN	0.88^D^	0.87^B^	2.16^AB^

**Table 3:** Least significant difference test for lesion size, difenoconazole and mancozeb mean relative growth rates (MRGR) between Mat-1 and Mat-2 isolates in the seven *A. alternata* populations collected from potato in China

	Lesion area	Difenconazole MRGR	Mancozeb MRGR
Mat-1	1.65^B^	0.90^A^	0.89^A^
Mat-2	2.17^A^	0.90^A^	0.89^A^

### Association between mancozeb and difenoconazole resistances

**Fig. 3:**
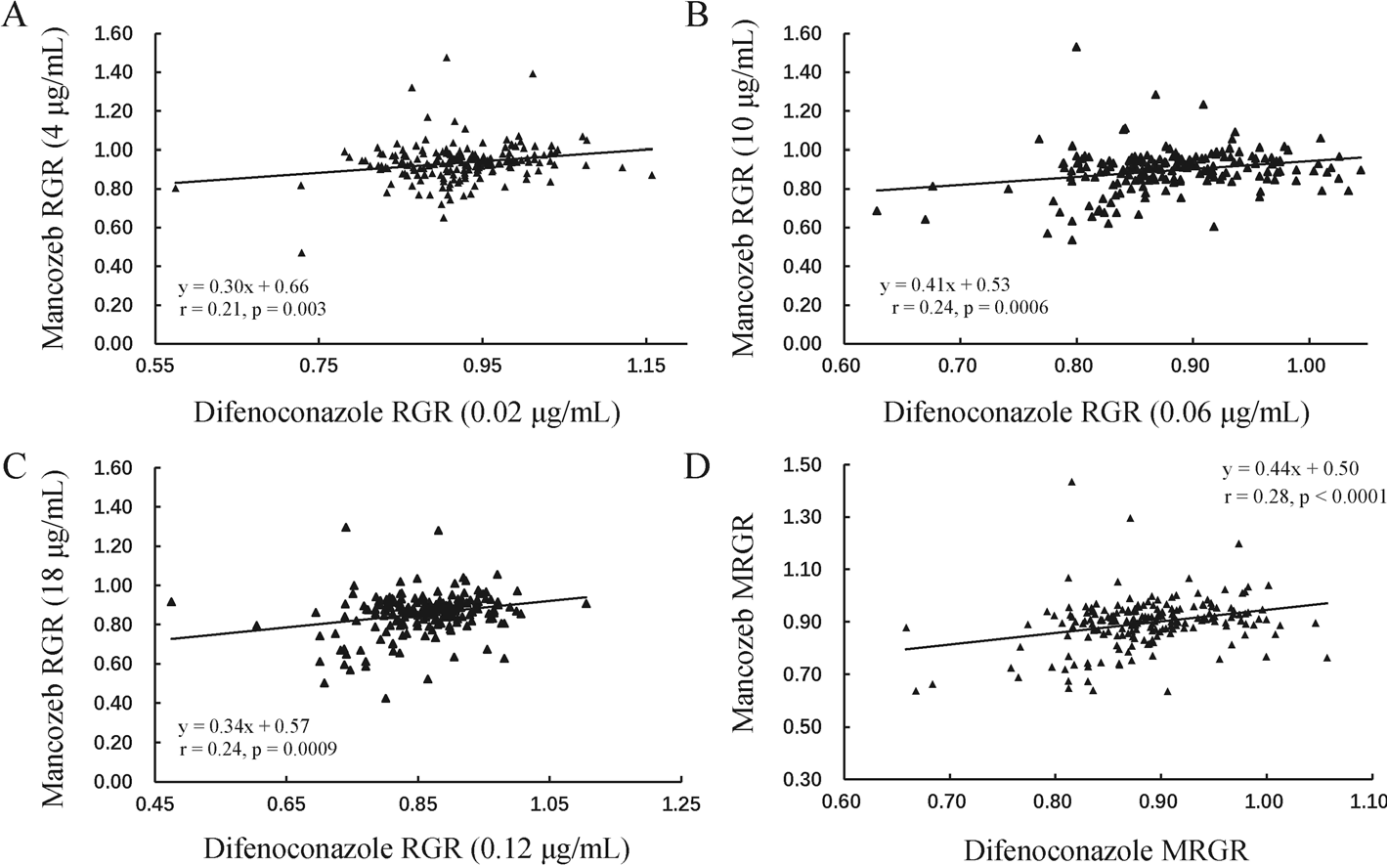
The correlation between mancozeb and difenoconazole tolerances in the *Alternaria alternata* isolates. **a** The correlation between mancozeb relative growth rate (RGR) in 4 μg/ml and difenoconazole relative growth rate (RGR) in 0.02 μg/ml. **b** The correlation between mancozeb RGR in 10 μg/ml and difenoconazole RGR in 0.06 μg/ml. **c** The correlation between mancozeb RGR in 18 μg/ml and difenoconazole RGR in 0.12 μg/ml. **d** The correlation between mancozeb MRGR and difenoconazole MRGR

There were positive and significant correlations between mancozeb and difenoconazole tolerances (Fig. 3). The correlation coefficient between mancozeb and difenoconazole tolerance at three concentrations from low to high was 0.21, 0.24 and 0.24 with the *p* values of 0.003, 0.0006 and 0.0009, respectively (Fig. 3a, b, c). The correlation coefficient between MRGRs of mancozeb and difenoconazole was 0.28 with a *p* value < 0.0001 (Fig. 3d).

### Multiple regression analysis between aggressiveness and fungicide tolerances

**Fig. 4:**
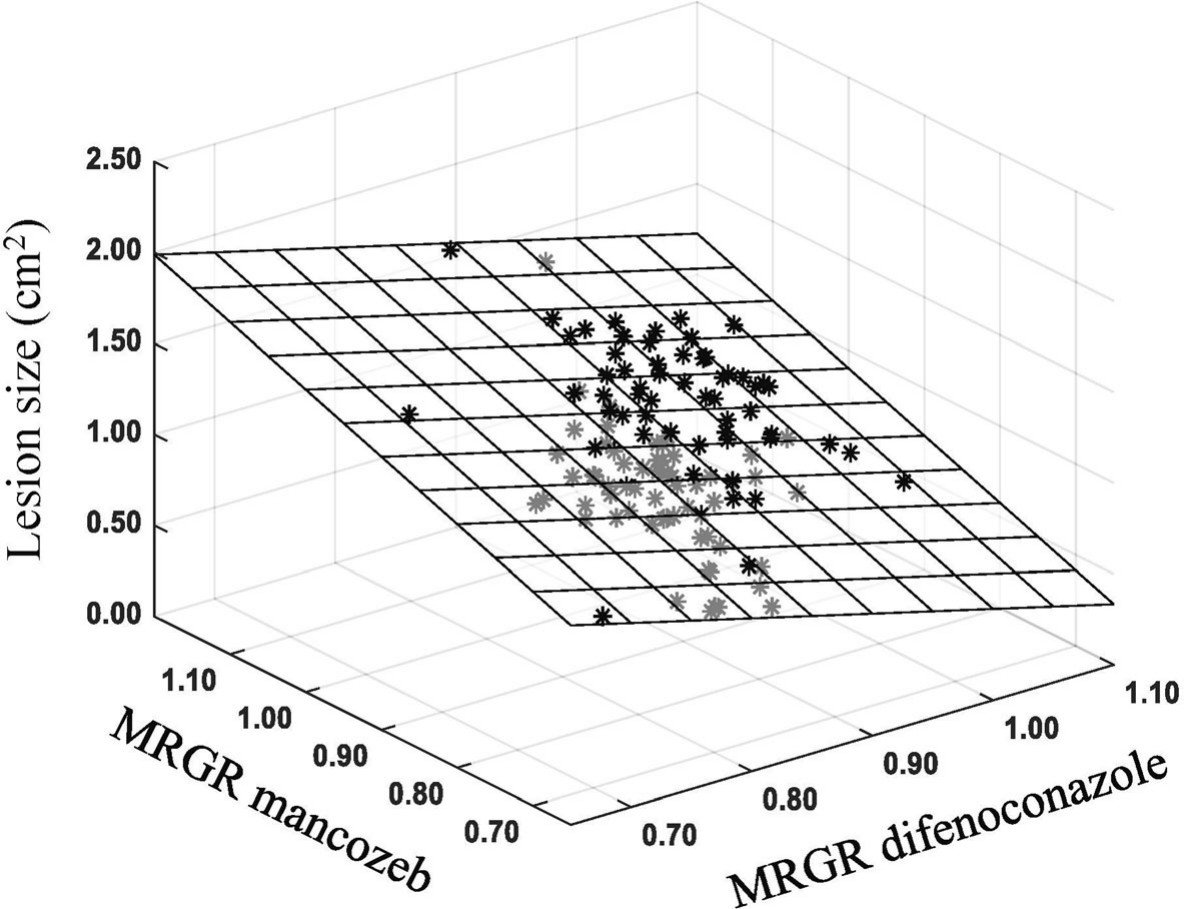
The multiple regression of the pathogenicity, mean mancozeb tolerance and the mean difenoconazole tolerance in *Alternaria alternata* populations. The square mesh is the equation fitting plane for the multiple regression analysis. The black stars are data above equation fitting plane, and gray stars are data below equation fitting plane

After removing outliers and incomplete data (missing one of the aggressiveness, mancozeb resistance or difenoconazole resistance values of an isolate), 142 isolates were included in the multiple regression analysis. Regression coefficients for mancozeb and difenoconazole tolerance were 1.62 and − 1.69, which are significant deviations from the null expectation and the intercept for the regression equation was 1.14. An F-test indicated that the data showed a good fit to the regression equation *y* = 1.62x_1_ − 1.69x_2_ + 1.14 (*p* < 0.0001, Fig. 4) where X_1_ and X_2_ represent the concentration of mancozeb and difenoconazole, respectively. Further analysis revealed that mancozeb tolerance was positively and significantly correlated with aggressiveness (r_141_ = 0.33, *p* = 0.0001) while difenoconazole resistance was negatively but not significantly correlated with aggressiveness (r_141_ = − 0.13, *p* = 0.1217).

## Discussion

Mancozeb and difenoconazole have been among the most frequently used fungicides to control plant diseases including potato early blight worldwide for some time. Despite this fact, no mancozeb resistant isolates were found in the study, consistent with previous results [12]. Significant growth inhibition was observed in the majority of isolates at low concentrations of the fungicide (Fig. 1). The same pattern of inhibition was found in difenoconazole. On the other hand, resistance to other fungicides such as pyraclostrobin, boscalid and fluxapyroxad can quickly be developed in the pathogen [26, 27]. Therefore, our results suggest that there is a low risk of developing mancozeb and difenoconazole resistance in *A. alternata* populations. This is likely associated with the chemical features of the two fungicides. Mancozeb is a site non-specific fungicide and evolution of resistance in the pathogen would involve changes in multiple genes across the *A. alternata* genome [13]. Though difenoconazole is a site-specific fungicide targeting *CYP51*, other mechanisms such as alteration in sterol biosynthesis pathways [28], increased active efflux by ABC transporters [29] and changes in cell membrane integrity and cell composition [30] also contributed to the building of drug resistance. Sequestration of the antifungal agents in cell membranes and reduced positive influx have been reported to play important role in the DMI resistance in many fungi [31] and may also involve the development of *A. alternata* resistance to difenoconazole. In addition, *A. alternata* has a broad host range including many grasses. A continuous influx of sensitive isolates from other wild hosts which are usually not exposed to the fungicides is likely to dilute selective pressures and prevent the build-up of resistance level in potato populations of *A. alternata* [32].

However, the finding of significant differences in mancozeb and difenoconazole tolerance among isolates and populations (Table 2) and skewing sensitivity distribution towards the right (Fig. 1, Additional file 1: Figure S1) suggest that a stepwise accumulation of tolerance to the fungicides might be occurring in the pathogen populations. This is the especial concern in difenoconazole. Due to the large number of isolates assayed, we only tested the sensitivity of the pathogen at three mancozeb and difenoconazole concentrations. Though this limitation does not allow us to calculate EC_50_, the half maximum effective concentration, accurately [33], rough estimates found that approximate 1% of *A. alternata* isolates (3 out of 234) had an EC_50_ value 10-fold greater than the baseline and therefore can be considered as candidate progenitors of future resistant genotypes [12, 34]. Therefore, caution should be taken in practice to prevent amplification in the frequency of these genotypes.

In the comparison of fungicide resistance among populations we found that *A. alternata* isolates from different locations tend to have similar mancozeb and difenoconazole tolerance rankings (Table 2). This motivated us to conduct a cross-resistance analysis for two fungicides differing in their mode of action. The finding of positive correlation between mancozeb and difenoconazole resistance in individual concentrations and combination clearly points to cross-resistance between the two fungicides (Fig. 3). Our finding is consistent with a previous result showing that *A. alternata* collected from tomato plants demonstrated cross-resistance of mancozeb with cyprodinil and tebuconazole, two other single-site fungicides in FRAC 2010 [12] and suggest cross-resistance to fungicides with different action modes might not be a rare event in *A. alternata* or other pathogens. Cross-resistance can occur when a fungicide selects for a gene which is linked to genes responsible for the development of resistance to other fungicides (so-called “genetic hitchhiking” [35, 36]). However, the cross-resistance between mancozeb and difenoconazole found in the current study is unlikely to be caused by a hitchhiking effect due to the complex mechanisms involved in mancozeb resistance. Genome-wide analysis uncovered 286 resistance determinants to mancozeb in yeast [17].

Instead, we hypothesize that mechanisms such as enhancing drug efflux and/or production of detoxifying metabolites limit the intercellular accumulation of fungicides. Fungi adopt many mechanisms including ATP binding cassette (ABC) transporters and major facilitator superfamily (MFS) transporters to export unwanted and/or toxic compounds such as fungicides [14]. For example, the analyses of knock-out and overexpression mutants revealed that a specialized ABC efflux transporter in *Grosmannia clavigera* conferred monoterpene resistance [37]. Similarly, the MFS transporter Mfs1 in *Botrytis cinerea* was found to mediate efflux of several fungicides as well as plant- and microbe-derived toxins [38]. It has been proposed that similar drug efflux mechanisms might exist in *A. alternata,* resulting in cross-resistance of the pathogen to several fungicides with different modes of action [12].

The observed cross-resistance could also be caused by the production of pathogen metabolites that destroy or modify the structures and functions of both natural and synthetic antimicrobials such as fungicides. Melanin is one of such metabolites. It is a ubiquitous pigment formed by the oxidative polymerization of phenolic compounds and can be produced by many plant and human pathogens [39–41]. Empirically, it has been documented that melanin plays an important role in pathogen’s adaptation, including in increasing their virulence and resistance to antimicrobial compounds [42, 43]. The loss of melanization has been reported to cause reduced virulence and increased fungicide sensitivity in *A. alternata* [44–46]. Recently, it was found that some response regulators such as SSK1 and SHO1 can simultaneously regulate the adaptation of *A. alternata* to environmental stresses including oxidation, osmotic pressure and fungicides [47]. Mutations in such response regulators might also contribute to the observed cross-resistance.

Fitness penalties have been proposed to be one of main mechanisms mitigating the evolution of fungicide resistance. Fungicide resistance, even caused by mutations in a single gene, involves genome-wide changes in gene expression [48, 49]. Fitness costs may be incurred in resistant mutations due to the disturbance of normal gene function or expression profiles [50, 51]. However, no fitness penalties were detected in the current study. Instead, multiple regression analysis showed that mancozeb tolerance of *A. alternata* was positively associated with aggressiveness (Fig. 4), suggesting fungicide resistant mutants also tend to have high ability to cause disease. This result is counter intuitive but consistent with previous publications where it was found that *A. alternata* isolates from tomato plants resistant to mancozeb were more aggressive [12] and *Z. tritici* isolates highly tolerant to cyproconazole also induced more disease symptom on susceptible wheat [52]. Like cross-resistance, positive associations between mancozeb tolerance and aggressiveness in *A. alternata* may also be attributed to enhancing drug efflux and/or production of detoxifying metabolites. Plant immune systems usually involve the production of compounds that have lethal or inhibitory effects on pathogens [53]. These immunity-associated compounds may share some structural or functional characteristics with mancozeb. *A. alternata* isolates having the ability to detoxify and/or export the compounds produced by host plants may also have the ability to detoxify and/or export mancozeb, leading to a simultaneous increase in aggressiveness and mancozeb tolerance [52]. Indeed, an experimental evolution study of antifungal drug resistance in *Saccharomyces cerevisiae* revealed that populations that gained drug resistance by the overexpression of the ABC transporter genes had higher fitness than the progenitor both in the presence and absence of fluconazole [54]. The fitness gains by melanin biosynthesis is another possibility due to its role in reducing the sensitivity of melanized cells to drugs and increasing aggressiveness by interfering with numerous host defense mechanisms [42, 44–46, 55].

## Conclusion

Results from this study have several important practical implications. The finding of skewing tolerance distributions to a higher level and significant differences in tolerance to both fungicides among isolates and populations suggest the accumulation of tolerance is occurring in the pathogen populations, particularly for difenoconazole to which 1% of isolates already show some evidence of resistance. The detection of cross-resistance among different classes of fungicides suggests that the mode of action alone may not be an adequate sole criterion to determine what components to use in the mixture and/or rotation of fungicides in agricultural and medical sects. Similarly, the occurrence of a positive association between aggressiveness and mancozeb tolerance suggests that intensive application of site non-specific fungicides might select for pathogens with both reduced sensitivity to fungicides and enhanced ability to cause diseases, thereby posing a greater threat to agricultural production and human health. In this case, the use of evolutionary principles in closely monitoring populations and the use of appropriate fungicide applications are important for effective use of the fungicides and durable infectious disease management [56, 57].

## Methods

### *Alternaria alternata* collection

*Alternaria alternata* isolates collected from seven potato fields located in Fujian (FJN), Heilongjiang (HLJ), Henan (HNN), Hubei (HBI), Inner Mongolia (IMG), Shandong (SDG) and Yunnan (YNN) provinces during the 2011 and 2012 potato growing seasons were previously genotyped with neutral SSR markers and PCR amplifications of mating types [24] and stored at − 80 °C in silica gels until use. A total of 234 genetically dinstint isolates were included in the study, representing 31, 31, 33, 28, 39, 32 and 40 isolates from FJN, HLJ, HNN, HBI, IMG, SDG and YNN, respectively. Among these, 119 isolates are Mat-1 and 115 isolates are Mat-2. Detailed information on pathogen collection, isolation, DNA extraction, SSR assay and mating type determination can be found in the previous publications [25, 58]. Briefly, infected leaves with typical early blight symptoms were collected randomly from potato plants separated by 1–2 m with only one infected leaf being sampled from each collection point (plant). After collection, individual leaves were immediately placed in separate sandwich bags to prevent cross infection and transferred within 24 h to the laboratory for pathogen isolation. One single-spore strain was isolated from each infected leaf. Genomic DNAs were extracted using a plant gDNA kit (Promega Biotech. Co. LTD., Beijing) and amplified with eight pairs of SSR primers and two pairs of mating type-specific primers in a total reaction volume of 25 μL using a 2720 thermal 163 cycler (Applied Biosystems, Foster City, California).

### Determination of fungicide tolerance

Before the fungicide experiment, all isolates were revived from long-term storage by growth on PDA plates for 6 days. Fungicide tolerance was tested at concentrations of 0, 4, 10 and 18 μg/ml mancozeb and 0, 0.02, 0.06 and 0.12 μg/ml difenoconazole, respectively. Many isolates did not grow when higher concentrations were used while growth rates of many isolates did not change when lower concentrations were used. The experimental test of fungicide resistance involved placing mycelial plugs (5 mm in diameter) taken from the margin of a growing colony onto 9-cm PDA plates supplemented with or without different concentrations of fungicides prepared from technical grade material. The plates were grouped into three separate batches (replicates) each corresponding to one of the three fungicide concentrations in each fungicide and laid out in a randomized complete block design (RCBD) using three replicates as described in previous publications [32, 56]. Controls (no fungicide) were included in each batch of plates. Media and inoculations for the entire experiment were made by the same person with all isolate-replicate combinations for a single fungicide concentration being assessed on the same day in a single incubator set to 24 °C. Plates were photographed daily between day two and six post-inoculation and colony areas were measured with the image analysis software Assess [59]. Thus, a total of 28,080 [234 isolates × 3 replicates × 4 treatments (3 fungicide concentrations + 1 control) × 5 measurements × 2 fungicides] data points were used to evaluate mancozeb and difenoconazole tolerance.

### Aggressiveness test

Aggressiveness was measured on detached leaves of the susceptible potato cultivar Favorita. Fully expanded leaves excised from Favorita plants grown in field for 8 weeks were placed on 1% water agar in petri dishes and then inoculated on the abaxial side with mycelial plugs (5 mm in diameter). Four detached leaves were inoculated with each isolate (four replicates). The petri dishes with detached leaves were arranged in CBD and maintained in 16-h days at 24 °C. Diseased leaves were photographed daily between day two and five post-inoculation and lesion areas were analyzed electronically with the image analysis software Assess [59].

### Data analysis

A logistic model based on measured colony sizes of pathogen isolates over the 6 days under each fungicide concentration was used to estimate growth rates [60]. The initial colony size at the point of inoculation (day one) was set to 0.2 cm^2^ (πr^2^ = 3.14 × 0.25^2^, here 0.35 is the radius of mycelial plugs) and the capacity of colony growth (K) for the logistic model was set to 63.6 cm^2^ (πr^2^ = 3.14 × 4.5^2^, here 4.5 is the radius of 9-cm petri dish). Mancozeb and difenoconazole tolerance was measured by the growth rate of isolates in the presence of the fungicide relative to that in the absence of the fungicide (RGR) [56, 61]. Mean relative growth rate (MRGR) of pathogen was calculated from the average of RGRs at three different fungicide concentrations. Fungal aggressiveness was estimated by lesion areas on the 5th day of post-inoculation on detached Favorita leaves. Frequency distributions of fungicide tolerances and aggressiveness of the fungal isolates were tabulated using a binning approach and each group was labeled with the upper boundaries of the corresponding bins. Analysis of variance of fungal aggressiveness and fungicides tolerances was performed using a general linear model implemented in SAS 9.4 (SAS Institute) by treating “fungicides concentrations” and “fungal mating type” as fixed effect and “populations” and “isolate” as random effects. Least significant differences [62] were used to compare fungal aggressiveness and fungicides tolerance among the seven *A. alternata* populations and between the two mating type groups across all seven populations. Associations of mancozeb tolerance with difenoconazole tolerance (RGR and MRGR respectively) were evaluated by simple linear correlation [63]. Multiple linear regression analysis implemented in Matlab [34] was used to evaluate the association among mancozeb tolerance, difenoconazole tolerance and aggressiveness of isolates using the model: *y* = b1x1 + b2x2 + c, where c is the intercept, y, x1 and x2 were lesion area, mancozeb tolerance and difenoconazole tolerance and b1 and b2 were regression coefficients for mancozeb and difenoconazole, respectively. Matlab [64] was also used to determine outliers which were discarded in parameter estimation and validation and to plot a figure showing the relationship among aggressiveness and fungicide resistance. The total number of isolates in each analysis was not identical due to some missing phenotypic characteristics data.

## Additional file

Additional file 1: **Figure S1.** The frequency distribution of fungicide tolerance in the *Alternaria alternata* isolates in different years. (TIFF 569 kb)

## Data Availability

The authors declare that the data obtained in this study are available upon request from the corresponding author (Jiasui Zhan, email: jiasui.zhan@fafu.edu.cn) to editorial board members, referees and readers.

## Abbreviations

ABC: ATP-binding cassette
DMI: demethylation inhibition
EBIS: ethylene bisisothiocyanate sulfide
FNN: Fujian
FRAC: Fungicide Resistance Action Committee
HBI: Hubei
HLJ: Heilongjiang
HNN: Henan
IMG: Inner Mongolia
MFS: Major facilitator superfamily
MRGR: Mean relative growth rate
RGR: Relative growth rate
SDG: Shandong
SSR: Simple sequence repeats
YNN: Yunnan
