# Supplementary figures and images for: Cross-resistance of the pathogenic fungus Alternaria alternata to fungicides with different modes of action
Source: BMC Microbiol. 2019 Sep 2;19:205. doi: 10.1186/s12866-019-1574-8 (PMC6720428; doi:10.1186/s12866-019-1574-8)

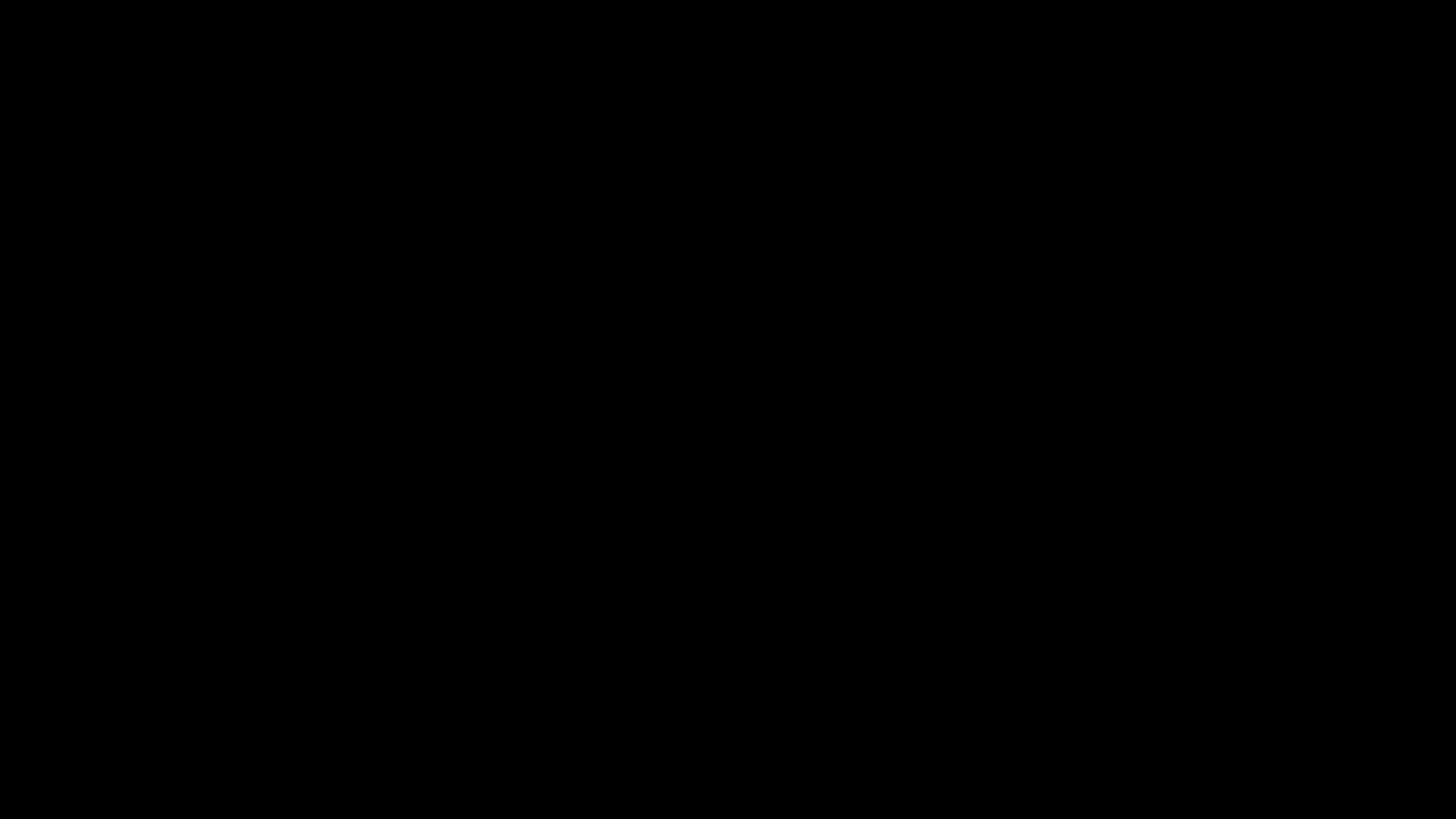

Supplement: Supplementary file 1 — Figure S1. The frequency distribution of fungicide tolerance in the Alternaria alternata isolates in different years. (TIFF 569 kb) [file 12866_2019_1574_MOESM1_ESM.tiff]
